# Efficacy of Leukocyte Platelet Rich Fibrin (L-PRF) Versus L-PRF with βeta Tri-calcium Phosphate (β-TCP) Bone Graft in Mandibular Impacted Third Molar Surgery: A Split-Mouth Double Blinded Randomized Control Study 

**DOI:** 10.30476/dentjods.2025.106480.2667

**Published:** 2026-09-01

**Authors:** George Samyo Stephenson, Deepak Abraham Pandyan, Satheesh Chandran, Deenadayalan Narasimman, Karthik Kattur Premkumar, Balamurugan Rajendran, Vijayalakshmi Pethuraj

**Affiliations:** 1 Dept. of Oral and Maxillofacial Surgery, Madha Dental College and Hospital, Chennai, India.; 2 Oral and Maxillofacial Surgeon and Oral Implantologist, Dept. of Dentistry, RYA MADRAS COSMO Foundation Hospital, Chennai, India.; 3 Orthodontist, Private Clinical Practitioner, Chennai, India.

**Keywords:** Platelet rich fibrin, βeta-tricalcium phosphate, Wound healing, Bone density

## Abstract

**Background:**

Mandibular third molar removal surgically exhibits severe pain, edema, limitation in mouth opening, impaired wound healing, and delayed bone formation postoperatively.

**Purpose:**

To compare the effectiveness of leukocyte platelet-rich fibrin (L-PRF) and L-PRF with β-Tricalcium phosphate (β-TCP) bone graft on postoperative outcomes after mandibular impacted third molar surgery.

**Materials and Method:**

This split-mouth double-blinded randomized controlled study was conducted on 100 patients, who presented for bilateral extraction of mandibular impacted third molars. Group I included 50 patients who were subjected to L-PRF, and group II included 50 patients who were subjected to L-PRF with β-TCP in the extracted socket after removal of the mandibular third molar. The surgical site bleeding was assessed intraoperatively. The pain, swelling, and trismus were evaluated on the 1^st^, 3^rd^, and 7^th^ postoperative days. Wound healing was evaluated on the 3rd and 7th postoperative days, and bone density was assessed on the 3rd postoperative month using cone beam computed tomography (CBCT).

**Results:**

Group II patients experienced reduced surgical site bleeding, less postoperative swelling, maximum mouth opening, and improved bone regeneration (*p* Value< 0.05). Group I patients exhibited faster wound healing and less postoperative pain (*p*< 0.05).

**Conclusion:**

The results of our study indicate that L-PRF combined with β-TCP remains an excellent and promising biocompatible material for achieving better postoperative outcomes compared to L-PRF alone.

## Introduction

Mandibular third molar removal can pose significant complications, including pain, edema, trismus, impaired wound healing, and dry socket. Long-term effects after removal cause migration of the cellular component leading to drastic resorption of the alveolar bone [ 1 - 3 ]. Repair of bone tissues involves mineralization and cellular functions, followed by the phase of remodeling to restore its original anatomical structure. Employment of various biological materials such as bone grafts, proteins, and barrier membranes aids in supporting the process of bone regeneration [ 3 ]. 

Platelet concentrates are highly beneficial for post-surgical outcomes due to their effectiveness in stimulating growth factors [ 4 ]. Leukocyte platelet-rich fibrin (L-PRF) is an autologous material that accelerates bone formation and promotes wound healing after third molar extraction [ 5 ]. However, L-PRF greatly impacts the density of the newly formed bone [ 6 ]. β-Tricalcium phosphate (β-TCP), an osteoplastic material, reduces the rate of bone resorption by 3.5 months post-extraction and in-creases its efficiency of bone regeneration by 6 months when loaded locally into the third molar socket [ 7 - 8 ]. β-TCP, owing to its superior properties of biocompatibility and biodegradability, has been considered as a material of high propensity for bone substitution [ 9 ]. 

In general, leukocytes in platelet-rich fibrin release enormous growth factors for a period of one week [ 10 ] to twenty-eight days [ 11 ] to promote angiogenesis, which eventually lays a biological matrix by supporting cell migration and cytokine release [ 12 ]. Pavani *et al*. [ 13 ] described the use of platelet-rich fibrin (PRF) as an adjunctive biological material in the management of bone defects. This signifies that β-TCP, being an osteoconductive material, when combined with L-PRF, may exert the effects of osteoinduction. However, the results remain controversial in the literature [ 14 ].

Hence, the present study aimed to compare the effects of L-PRF and L-PRF with β-TCP on surgical site bleeding, postoperative pain, swelling, trismus, wound healing, and bone regeneration after mandibular impacted third molar surgery. 

## Materials and Method

A split-mouth double-blinded randomized controlled study was conducted on 100 patients who reported to the Department of Oral and Maxillofacial Surgery at Madha Dental College and Hospital, Chennai, India, for mandibular impacted third molar removal. The study protocols were postulated, and ethical clearance was obtained from the ethical committee of our institution (MDCH/IEC/2020/06, Madha Dental College and Hospital, Chennai, India). An informed consent form in the vernacular language was signed by all subjects included in the study. Study inclusions were bilateral impacted mandibular third molars in the age group of 20-40 years. Exclusions were periodontally compromised individuals, patients with bleeding disorders, pregnant and lactating patients, patients with known systemic diseases, patients with known allergies, and patients with metabolic disorders.

The sample size was calculated using the statistical software G. power 3.1.9.2. Based on the effect size 0.32, power 90%, confidence 95%, the sample size was determined to be 100 patients (50 in each group). Double blinding was performed in this study, where both the patients and the investigator were unaware of the materials placed inside the third molar socket after mandibular third molar surgery. The patients included in this study were allocated into groups through a computer-generated randomization method (Random.org). The allocation concealment was established before the initiation of treatment and performed by a third person who was not involved in the study.

### Groups

Group I included 50 patients who were placed with prepared L-PRF into the extracted socket, and group II included 50 patients who were placed with L-PRF along with β-TCP bone graft locally into the extracted socket after mandibular third molar surgery. 

### Procedure

**Figure 1:**
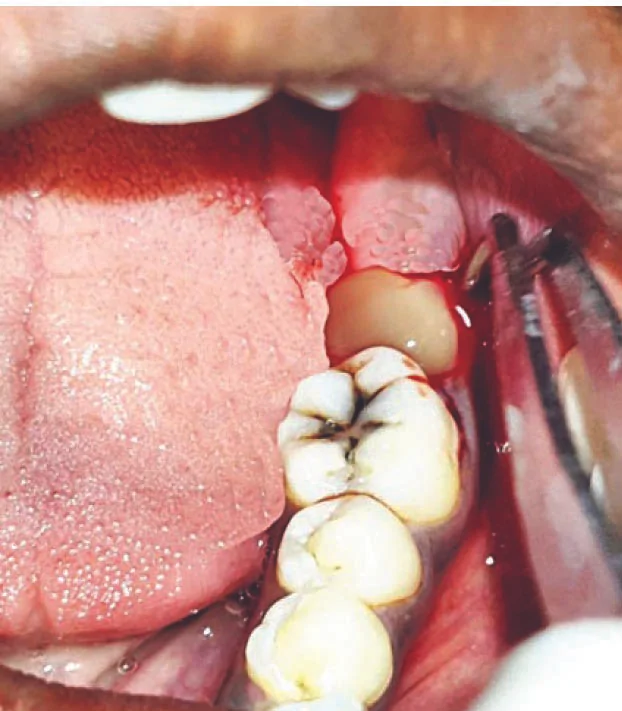
Placement of leukocyte platelet rich fibrin (L-PRF) after removal of mandibular third molar

**Figure 2:**
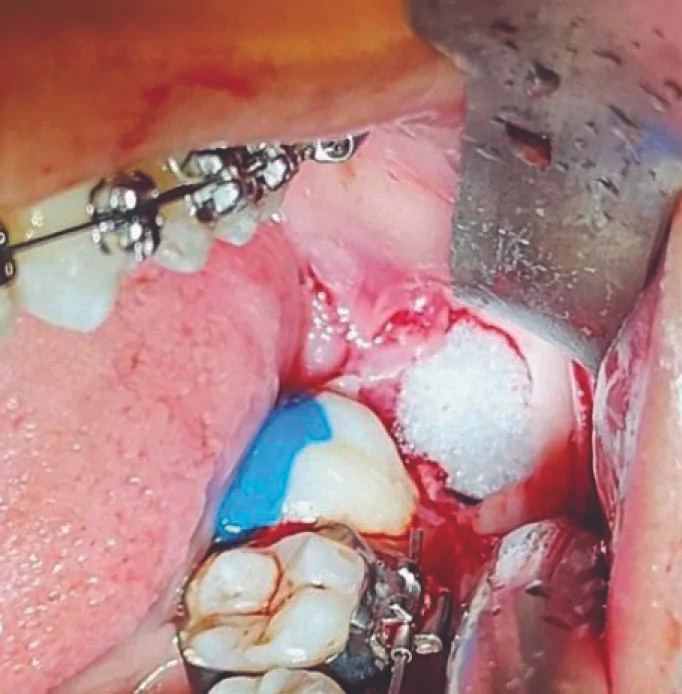
Placement of leukocyte platelet rich fibrin (L-PRF) with β-Tricalcium phosphate (β-TCP) after removal of mandibular third molar

The procedure was performed by a single experienced oral surgeon and carried out under an aseptic environment. Preoperative site preparation was performed using a 5% povidone-iodine solution. Inferior alveolar nerve block was administered with 2% lidocaine and 1in 80,000 adrenaline. A Ward’s incision was placed using a no15 scalpel in both groups. A full-thickness mucoperiosteal flap was raised with a Molt’s periosteal elevator to expose the third molar. Bone guttering was performed using #702 bur under copious saline irrigation. The tooth was elevated or sectioned and retrieved using forceps. The socket was carefully examined for remnants and then thoroughly irrigated with a saline. The prepared L-PRF was placed in the extracted socket of group I patients (Figure 1), and L-PRF with β-TCP bone graft (Ammdent Ostoden bone graft material, Amrit Chemicals and Minerals Agency, India) was placed into the extracted socket of group II patients (Figure 2). The extracted sockets were then closed primarily using 3-0 silk sutures. Post-operative instructions were given to the subjects of both groups. All the participants were prescribed with antibiotics and analgesics post-surgery for 5 days. 

### Preparation of leukocyte platelet-rich fibrin

A tourniquet was tied on to the patient's arm. About 9ml of venous blood was withdrawn and collected in a sterile tube without any anticoagulants. The tube was then centrifuged in a (Medico Plus, REMI, India) centrifuge machine at 2700rpm for 12 minutes. The resultant product consisted of acellular plasma at the top, L-PRF in the middle, and RBCs at the bottom. The L-PRF was then meticulously separated from the residual components and stored in a sterile container for its use in the extracted socket after removal of the mandibular third molar. 

### Follow-up and assessments

Patients included in this study were evaluated for surgical site bleeding intraoperatively using Fromme’s scale, which ranges from 0 to 5 [ 15 ]. Pain, swelling, and trismus were evaluated on the 1^st^, 3^rd^, and 7^th^ postoperative days. Evaluation of pain was performed using a 10 cm visual analogue scale (VAS). The score ranges between 0 and 10, where 0 represents no pain, 5 represents moderate pain, and 10 represents the worst possible pain. Facial swelling was assessed using the craniometric method by measuring three distinct landmarks in millimeters (mm). The first landmark measured from the lateral canthus of the eye to the angle of the mandible was represented as S1. The second landmark measured from the tragus of the ear to the corner of the mouth was represented as S2, and the third landmark measured from the tragus of the ear to the soft tissue pogonion was represented as S3. The average of all three landmarks was represented as swelling S. Mouth opening was recorded in millimeters (mm) by measuring the distance between the upper and lower central incisors using a string. Wound healing was evaluated on the 3^rd^ and 7^th^ postoperative day using modified Landry’s wound healing score (Table 1). An outcome of 1 signifies poor wound healing, and 5 signifies excellent wound healing. Bone density was assessed using cone beam computed tomography (CBCT) in grey scale values (GSV) at the 3^rd^ month postoperatively.

**Table 1:** Modified Landry’s wound healing score

Healing Score	Characteristics
1. Very poor	● Tissue colour: ≥50% of gingiva red
	● Response to palpation: bleeding
	● Granulation tissue: present
	● Suppuration present
	● Presence of Alveolar osteitis
2. Poor	● Tissue colour: ≥50% of gingiva red
	●Response to palpation: bleeding
	● Granulation tissue: present
3. Good	● Tissue colour: ≥25% and ≤50% of gingiva red
	● Response to palpation: no bleeding
	● Granulation tissue: none
4. Very good	● Tissue colour: < 25% and ≤50% of gingiva red
	● Response to palpation: no bleeding
	● Granulation tissue: none
5. Excellent	● Tissue colour: all tissues pink
	● Response to palpation: no bleeding
	● Granulation tissue: none

### Statistical Analysis

Data analysis was performed using IBM SPSS statistics V23 software. An independent t-test was employed to compare the parameters of pain, swelling, trismus, surgical site bleeding, wound healing, and bone density between groups with mean differences and standard deviations. *p* Value< 0.05 was considered statistically significant. 

## Results

**Figure 3:**
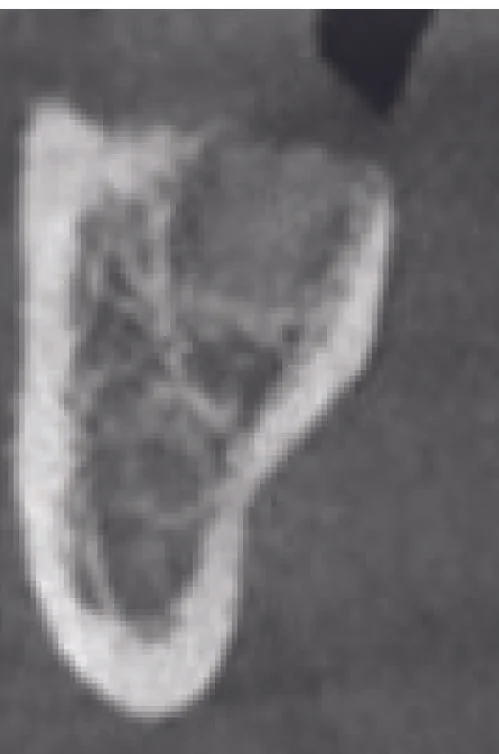
Bone regeneration at 3^rd^ month in leukocyte platelet rich fibrin (L-PRF) group

**Figure 4:**
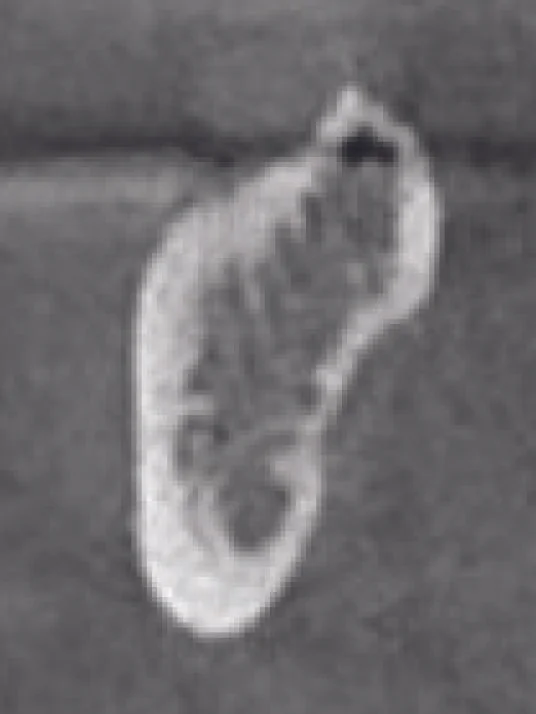
Bone regeneration at 3^rd^ month in leukocyte platelet rich fibrin (L-PRF) with β-Tricalcium phosphate (β-TCP) group

The current study included 100 patients who required bilateral removal of mandibular impacted third molars. Among 100 patients, 66 were male and 34 were female with an average age of 29 years. The obtained data were recorded in an excel worksheet and statistically evaluated using an independent t-test through SPSS software (Table 2, Figures 3-4). The participants in this study were retained until the completion of the study, and none were withdrawn during the research process. 

**Table 2:** Independent t test of statistical significance for surgical site bleeding, pain, swelling, trismus, wound healing and bone density between Group I and Group II

Parameters	Group I Mean (SD)	95% Confidence Interval (Lower bound, Upper bound)	Group II Mean (SD)	95% Confidence Interval (Lower bound, Upper bound)	*p* Value
Surgical site bleeding	2.26 (0.44)	2.138, 2.382	0.33 (0.47)	0.200, 0.460	0.00
Pain (vas)					
Day 1	4.16 (1.76)	3.672, 4.648	6.24 (1.56)	5.808, 6.672	0.04
Day 3	2.96 (1.22)	2.622, 3.298	4.84 (1.32)	4.474, 5.206	0.02
Day 7	1.06 (0.62)	0.888, 1.232	2.96 (1.17)	2.636, 3.284	0.00
Swelling (mm)					
Day 1	15.81 (0.71)	15.613, 16.007	14.67 (0.84)	14.437, 14.903	0.03
Day 3	14.96 (0.62)	14.788, 15.132	13.46 (0.66)	13.277, 13.643	1.12
Day 7	14.08 (0.48)	13.947, 14.213	12.61 (0.40)	12.499, 12.721	0.04
Mouth opening (mm)					
Day 1	19.26 (5.17)	17.827, 20.693	23.02 (4.25)	21.842, 24.198	0.02
Day 3	22.86 (4.66)	21.568, 24.152	28.76 (3.84)	27.696, 29.824	0.01
Day 7	26.93 (4.06)	25.805, 28.055	33.01 (2.82)	32.228, 33.792	0.01
Wound healing					
Day 3	4.72 (1.42)	4.326, 5.114	2.67 (1.78)	2.1777, 3.163	0.00
Day 7	5.01 (0.43)	4.891, 5.129	3.48 (0.75)	3.272, 3.6888	0.00
Bone Density (GSV)					
Month 3	117.2 (18.1)	112.183, 122.217	150.7 (23.1)	144.297,157.103	0.01

**Figure 5:**
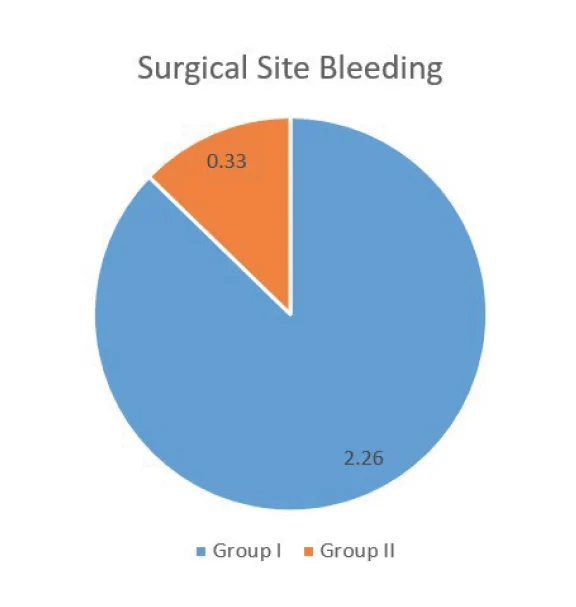
Surgical site bleeding between leukocyte platelet rich fibrin (L-PRF) and L-PRF with β-Tricalcium phosphate (β-TCP) groups

The intraoperative surgical site bleeding was comparatively reduced in group II patients compared to group I patients, with a mean difference of 0.33 (0.47) in group II and 2.26 (0.44) in group I, which showed a statistical significance of *p*< 0.05, respectively (Figure 5). 

**Figure 6:**
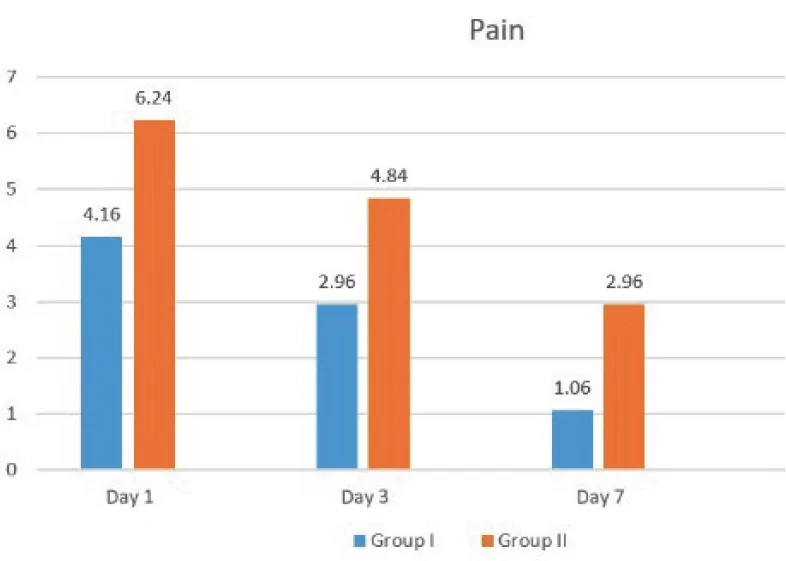
Postoperative outcomes on pain between leukocyte platelet rich fibrin (L-PRF) and L-PRF with β-Tricalcium phosphate (β-TCP) groups evaluated at 1^st^, 3^rd^ and 7^th^ days

The pain intensity in group I was comparatively less on all three postoperative days when compared to group II patients, with a mean difference of 4.16 (1.76) on day 1, 2.96 (1.22) on day 3, and 1.06 (0.62) on day 7, which showed a statistical significance of *p*< 0.05, respectively (Figure 6). 

**Figure 7:**
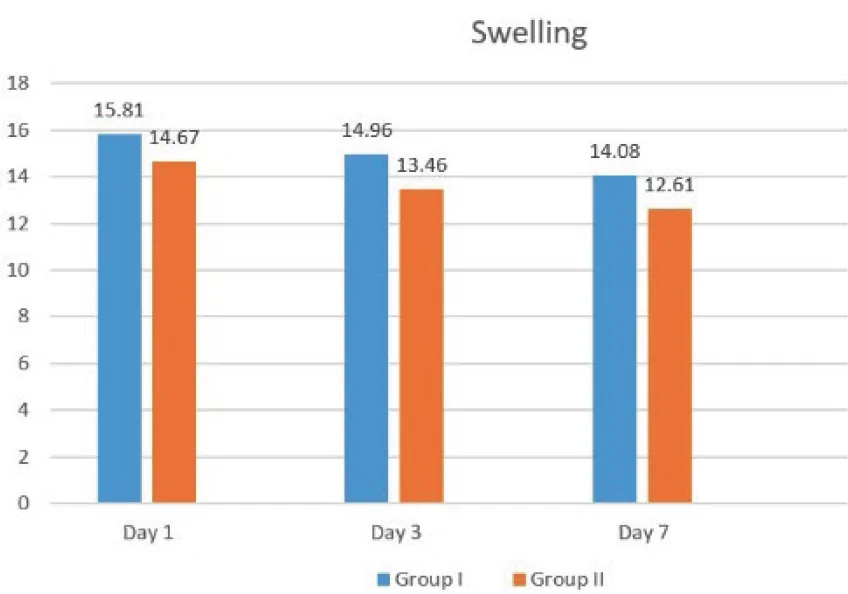
Postoperative outcomes on swelling between leukocyte platelet rich fibrin (L-PRF) and L-PRF with β-Tricalcium phosphate (β-TCP) groups evaluated at 1^st^, 3^rd^ and 7^th^ days

**Figure 8:**
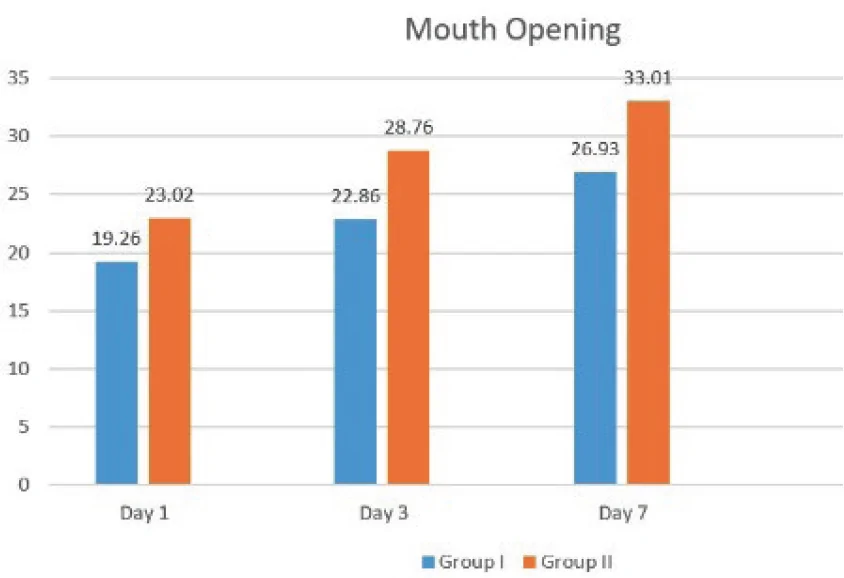
Postoperative outcomes on mouth opening between leukocyte platelet rich fibrin (L-PRF) and L-PRF with β-Tricalcium phosphate (β-TCP) groups evaluated at 1^st^, 3^rd^ and 7^th^ days

The amount of swelling was reduced in group II patients on the 1^st^ and 7^th^ postoperative days when compared to group I patients, with a mean difference of 14.67 (0.84) on day 1 and 12.61 (0.40) on day 7, which showed a statistical significance of *p* < 0.05, respectively (Figure 7). Mouth opening was improved in group II patients on all three postoperative days when compared to group I patients, with a mean difference of 23.02 (4.25) on day 1, 28.76 (3.84) on day 3, and 33.01 (2.82) on day 7, which showed a statistical significance of *p*< 0.05, respectively (Figure 8). 

**Figure 9:**
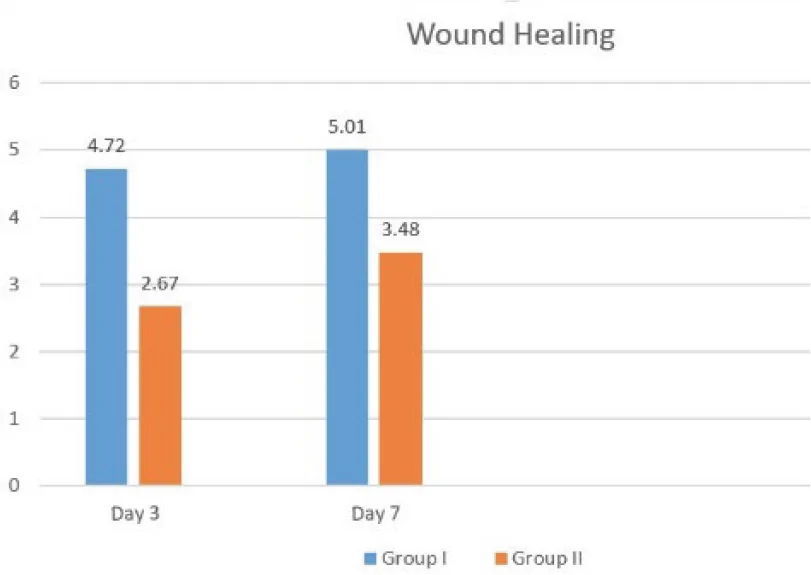
Postoperative outcomes on wound healing between leukocyte platelet rich fibrin (L-PRF) and L-PRF with β-Tricalcium phosphate (β-TCP) groups evaluated at 3^rd^ and 7^th^ days

The patients in group I exhibited better soft tissue healing compared to those in group II, with a mean difference of 4.72 (1.42) on day 3 and 5.01 (0.43) on day 7, which showed a statistical significance of *p*< 0.05, respectively (Figure 9). 

**Figure 10:**
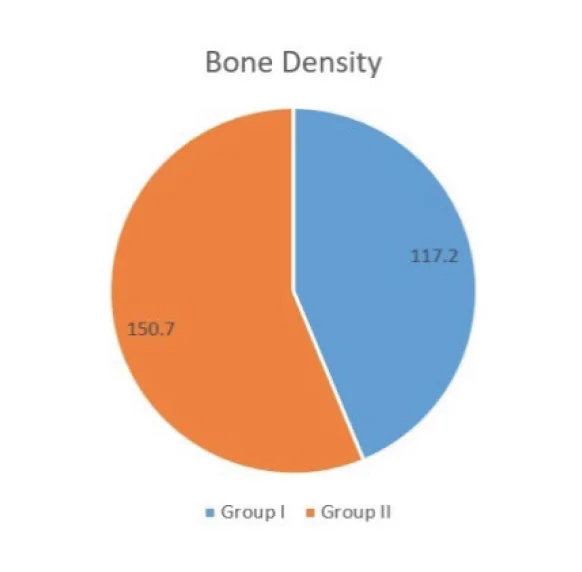
Postoperative outcomes on bone density between leukocyte platelet rich fibrin (L-PRF) and L-PRF with β-Tricalcium phosphate (β-TCP) groups evaluated at 3^rd^ month

The mean preoperative bone density was 54.9 (15.9) in group I patients and 50.9 (18.6) in group II patients. At the 3-month follow up, the mean bone density was 117.2 (18.1) in group I patients and 150.7 (23.1) in group II patients, which showed a statistical significance of *p*< 0.05 for group II patients, respectively (Figure 10). 

## Discussion

The study was to evaluate the efficacy of L-PRF and LPRF combined with β-TCP on postoperative complications after mandibular impacted third molar surgery.

Removal of mandibular third molars is a routine surgical procedure performed by clinicians in day-to-day practice, which results in numerous postoperative outcomes. The most profound entity experienced during third molar extraction was surgical site bleeding. Nourwali I. [ 2 ] emphasized that extraction sockets placed with PRF showed high-frequency bleeding at the 1^st^ hour postoperatively, compared to other postoperative periods. In our study, extraction sockets placed with a combination of L-PRF and β-TCP showed no surgical site bleeding when compared to the L-PRF group, with a statistical significance of *p*< 0.05. 

The current study assessed pain, swelling, and trismus at 1^st^, 3^rd^ and 7^th^ postoperative days. Singh *et al*. [ 16 ] reported that the PRF group experienced reduced pain compared to their control group. Trybek *et al*. [ 17 ] suggested that pain intensity was lower with PRF at 6 hours, 1^st^, and 3^rd^ days after surgery. Group I (L-PRF) patients of our study showed a drastic reduction in pain compared to group II (L-PRF with β-TCP), with *p*< 0.05. On the contrary, Gulsen and Senturk [ 18 ] showed statistically insignificant results on pain with the use of PRF when evaluated at 6 hours, 12 hours, 1^st^, 2nd, 3^rd^, and 7^th^ postoperative days. 

In the current study, group II patients experienced less edema on the 1^st^ and 7^th^ postoperative days (*p*< 0.05). However, Ozgul *et al*. [ 19 ] found a decrease in swelling in the PRF group only at a landmark measured from the tragus of ear to the corner of the mouth when evaluated on the 1^st^, 3^rd^, and 7^th^ postoperative days. Trybek *et al*. [ 17 ] reported no significant differences in the reduction of swelling with PRF when evaluated on the 1^st^, 2nd, and 7^th^ postoperative days, which showed a positive association with group I patients of our study. In terms of trismus, Uyanik *et al*. [ 20 ] observed a reduction in trismus with PRF only on day 1. Trybek *et al*. [ 17 ] found that the trismus was lower with the use of PRF on the 1^st^, 2nd, and 7^th^ postoperative days. Whereas, in our study, only group II patients showed a significant improvement in mouth opening compared to group I patients (*p*< 0.05). 

In the present study, wound healing was evaluated on the 3^rd^ and 7^th^ postoperative days in the two groups. Patients in group I experienced faster wound healing compared to those in group II, with a statistical significance of *p*< 0.05. The results of our study showed a positive correlation with those of Daugala *et al*. [ 21 ] who reported accelerated wound healing in the PRF group within 4 weeks. In contrast, Sunil *et al*. [ 22 ] emphasized that the infusion of L-PRF with β-TCP accelerated soft tissue healing compared to L-PRF alone in extraction sockets, as evaluated on the 3^rd^, 7^th^, and 14th postoperative days. Biomaterials such as allografts, xenografts or alloplasts, when placed into the extraction socket, tend to delay the process of wound healing [ 23 - 24 ] when compared to L-PRF. While L-PRF regenerates the surrounding tissues by enhancing cellular chemo-attraction, angiogenesis, and proliferation of epithelial cells, leading to complication-free wound healing [ 25 ].

The primary requisites of any bone graft must show activities of both osteoconduction and osteoinduction [ 22 ]. These bone grafts activate the progenitor cells, leading to cellular proliferation and differentiation, which eventually form a stable base for new bone formation [ 26 ]. PRF, when placed into the extracted socket, undergoes degranulation within 10 minutes of achieving hemostasis, which in turn secretes approximately 90% of its growth factors within one hour [ 27 ]. Resorption of PRF occurs approximately within 7 to 10 days [ 28 ]. β-TCP enhances L-PRF activity by providing a scaffold for bone regeneration and promoting osteoblast activity, leading to faster and more robust bone formation. β-TCP acts as a biocompatible and osteoconductive material, guiding new bone growth. While L-PRF provides growth factors and proteins that stimulate cell activity and accelerate the healing process.

In our study, group II patients exhibited faster bone formation at the 3-month follow-up compared to group I patients, which showed a direct correlation with the findings of Park *et al*. [ 29 ]. Kim *et al*. [ 30 ] signifies that PRF in combination with β-TCP yielded 8% to 10% rapid bone formation in the 2nd week, and Sunil *et al*. [ 22 ] resulted in a significant bone increment within the 3^rd^ week. These results significantly proved that PRF combined with β-TCP reduced the longer duration required for bone regeneration and risks associated with harvesting autologous bone grafts [ 31 ]. In general, Claflin *et al*. [ 32 ] and Jahangiri *et al*. [ 33 ] reported a 5- to 6-month duration to have a satisfactory bone formation after third molar surgery. In contrast to the above findings, the present study observed a satisfactory bone density within 3 months.

Remarking the shortcomings of our study, the type and size of the extracted socket for each participant were not determined before the placement of grafts. The amount of bone regeneration assessed at 3^rd^ postoperative month was adequate in our study, however; evaluations carried out at the 6th month and 1 year may yield improved bone formation. 

## Conclusion

Although L-PRF group had better reduction in postoperative pain and faster wound healing, L-PRF with β-TCP group showed minimal surgical site bleeding and a drastic reduction in swelling, with significant improvement in mouth opening, and promoted rapid bone regeneration. Hence, L-PRF combined with β-TCP remains an excellent and promising biocompatible material for achieving better postoperative outcomes compared to L-PRF alone.
